# Job vacancies at the European Centre for Disease Prevention and Control

**DOI:** 10.2807/1560-7917.ES.2019.24.44.1910311

**Published:** 2019-10-31

**Authors:** Eurosurveillance editorial team

**Affiliations:** 1 European Centre for Disease Prevention and Control (ECDC), Stockholm, Sweden

The European Centre for Disease Prevention and Control (ECDC) invites applications for the following positions:

 

- **Head of Unit Public Health Functions**

The application deadline expires on 22 Nov 2019.

- **Scientific Officer Infectious Diseases Epidemiology**

The application deadline expires on 26 Nov 2019. 

 

For more information, please visit the ECDC website: 

https://ecdc.europa.eu/en/work-us/vacancies?f%5B0%5D=deadline_date%3A1

